# Plasma essential fatty acid on hospital admission is a marker of COVID-19 disease severity

**DOI:** 10.1038/s41598-023-46247-0

**Published:** 2023-11-03

**Authors:** Vera C. Mazurak, Irma Magaly Rivas-Serna, Sarah R. Parsons, Md Monirujjaman, Krista E. Maybank, Stanley K. Woo, Oleksa G. Rewa, Andrew J. Cave, Caroline Richard, M. Thomas Clandinin

**Affiliations:** 1 Division of Human Nutrition, Department of Agricultural, Food and Nutritional Science, University of Alberta, Edmonton, T6G 2P5 Canada; 2 Department of Critical Care Medicine, Faculty of Medicine, University of Alberta, Edmonton, Canada; 3 Department of Family Medicine, University of Alberta, Edmonton, T6G 2P5 Canada; 4 Department of Medicine, University of Alberta, Edmonton, T6G 2P5 Canada

**Keywords:** Lipidomics, Cytokines, Chemokines, Interferons, Interleukins, Tumour-necrosis factors, Predictive markers, Biochemistry, Fatty acids, Phospholipids

## Abstract

It is important for allocation of resources to predict those COVID patients at high risk of dying or organ failure. Early signals to initiate cellular events of host immunity can be derived from essential fatty acid metabolites preceding the cascade of proinflammatory signals. Much research has focused on understanding later proinflammatory responses. We assessed if remodelling of plasma phospholipid content of essential fatty acids by the COVID-19 virus provides early markers for potential death and disease severity. Here we show that, at hospital admission, COVID-19 infected subjects who survive exhibit higher proportions of C20:4n-6 in plasma phospholipids concurrent with marked proinflammatory cytokine elevation in plasma compared to healthy subjects. In contrast, more than half of subjects who die of this virus exhibit very low C18:2n-6 and C20:4n-6 content in plasma phospholipids on hospital admission compared with healthy control subjects. Moreover, in these subjects who die, the low level of primary inflammatory signals indicates limited or aberrant stimulation of host immunity. We conclude that COVID-19 infection results in early fundamental remodelling of essential fatty acid metabolism. In subjects with high mortality, it appears that plasma n-6 fatty acid content is too low to stimulate cellular events of host immunity.

## Introduction

Infection with COVID-19 is accompanied by a dysregulated immune response that manifests early in the respiratory tract and has a high risk of death^1^. Morbidity of COVID-19 infection is attributed to respiratory failure and thrombus formation in major organs^2^. Some patients develop “Long COVID” symptoms that persist after the acute episode^3^.

Host immunity and accompanying inflammation is driven by signals derived from linoleic acid (C18:2n-6) and arachidonic acid (C20:4n-6)^4^. Essential fatty acid deficiency impacts immunological responses of both innate and acquired immunity to infection^5,6^. Severe disease and death are due to failure of nonspecific first-line defense mechanisms. COVID-19 infected patients with severe disease exhibit reduction in natural killer cell number and function, resulting in decreased clearance of infected cells and elevation of inflammation markers of tissue damage^7^.

Phospholipase A_2_, phosphatidylserine specific phospholipase and levels of prostaglandin E_2_ and F_2α_ are elevated after COVID-19 infection^8^. All types of immune cells constitutively express 5-lipoxygenase pathways and phospholipase A_2_ to enable leukotriene and oxylipin synthesis from C20:4n-6 containing phospholipid within seconds of immune stimuli. Leukotrienes regulate airway response and other immune responses and a deficit of leukotriene synthesis increases susceptibility to infection^5^. Interleukin (IL)-6, IL-2, IL-7, granulocyte-colony stimulating factor (G-CSF), Interferon (IFN)-ɣ, monocyte chemoattractant protein (MCP)-1; macrophage inflammatory protein (MIP)-1-α and tumor necrosis factor (TNF)-α orchestrate symptom progression^9–13^.

Viral trafficking, assembly, and release of viral particles requires phospholipid. Respiratory viruses infect the epithelium taking over cellular metabolism to support viral replication and production of a phospholipid coat by catabolizing host cell membrane and available plasma phospholipid, playing a critical role in coronavirus propagation^1^. Structural analysis of coronavirus spike protein also reveals that linoleic acid populates the spike protein binding site, suggesting a specific requirement by this virus for C18:2n-6 for replication and virus survival^14^. The overwhelming focus in recent literature relating essential fatty acids to COVID-19 infection is on potential anti-inflammatory benefits of n-3 fatty acids. No study has clearly associated n-6 and n-3 fatty acid status with outcomes, which could be relevant at a later stage of infection when host immunity is active. Lipidomic analysis after COVID-19 infection has been conducted but no specific observations defining risk or prognosis have been reported.

Demands of the replicating virus for essential fatty acid may compromise host immunity^1^, while remodelling plasma and respiratory epithelial fatty acid utilization to provide both essential n-6 and non-essential fatty acids for new virus. This demand by the virus for n-6 fatty acid is in direct competition with the need and role for C18:2n-6 and C20:4n-6 in initiating host immunity to contain viral replication. The objective of this study is to assess if the patient’s amount of available plasma n-6 fatty acid (i.e. low n-6 fatty acid status) appears to compromise initiation of host immunity. Plasma status could be an early plasma marker for disease severity and risk of death. This knowledge could remarkably affect treatment during the initial days of hospitalization and potentially disease outcome.

## Results

Subjects (n = 217) admitted to hospital for treatment of COVID-19 infection were approximately 5–7 days after infection. Approximately 70% of these subjects (n = 151) exhibited blood oxygen saturation less than 92% and were given supplemental oxygen (O_2_ group). Sixty-six subjects exhibited blood oxygen levels above 92% after admission to hospital and did not require oxygen (No O_2_ group). Forty of the 217 patients died during subsequent days.

**Figure 1:**
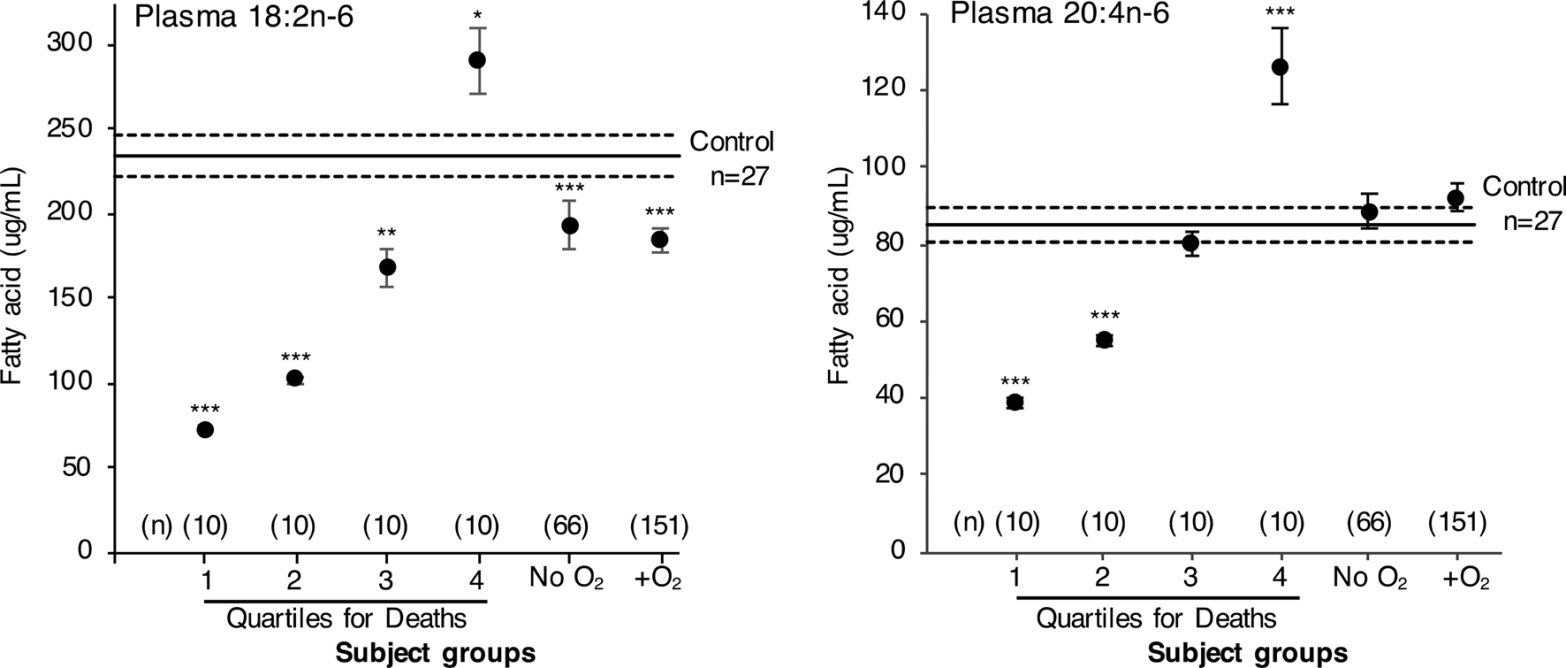
Quantitative amount of C18:2n-6 and C20:4n-6 in plasma. COVID-19 infected subjects on hospital admission are compared with control subjects. Values are expressed as μg of fatty acid/ml of plasma. Mean (–) ± SE (–---) is illustrated for healthy control subjects. Significant differences compared to control subjects were determined by analysis of variance and are indicated as; *p < 0.02, **p < 0.01, ***p < 0.001.

**Figure 2:**
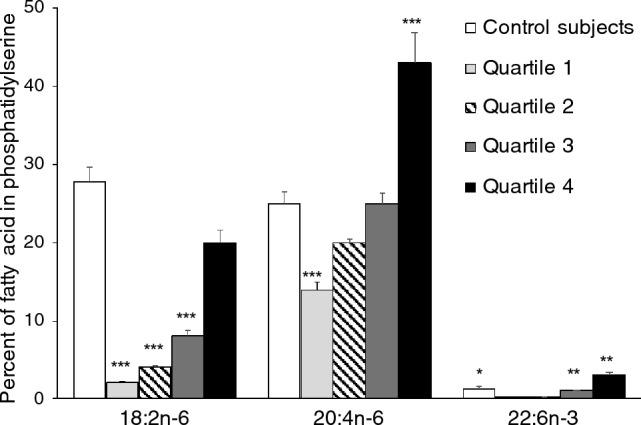
Essential fatty acid composition of phosphatidylserine in COVID-19 infected subjects who died. Values (mean ± SE) are illustrated as a percent of total fatty acids in phosphatidylserine. Significant differences compared to control subjects for each fatty acid were determined by analysis of variance and are indicated as; *p < 0.02, **p < 0.01, ***p < 0.001.

For all COVID-19 infected subjects, analysis of the n-6 and n-3 essential fatty acid composition (i.e. %w/w) of the total plasma phospholipid fraction (Table 1) revealed that COVID-19 infection results in significant decrease in C18:2n-6 and increase in C20:4n-6 composition in plasma phospholipids on day one of hospital admission compared to uninfected control subjects. Change in balance between essential fatty acids is characteristic in the major phospholipids, phosphatidylcholine and phosphatidylethanolamine which represent 92.5 ± 0.2% and 2.1 ± 0.1% of plasma phospholipids, respectively. Shift in balance toward synthesis of proinflammatory essential fatty acid is also apparent in the ratio of C18:2n-6 to C20:4n-6 representing an index that is characteristically decreased after infection. This ratio is lowest in subjects with more severe persisting respiratory symptoms and those that develop Long-COVID (Table 1). When the quantity of the total plasma content (µg/ml) of C18:2n-6 or C20:4n-6 was used to separate subjects who died into quartiles, it was revealed that 70% of subjects who died had very low total content of C18:2n-6 and C20:4n-6 in the plasma phospholipid on day one (Fig. 1) compared to normal control subjects or surviving COVID-19 infected subjects. Phosphatidylserine represents 0.8 ± 0.1% of the total plasma phospholipids. Lower 18:2n-6 in phosphatidylserine in COVID-19 infected patients compared to control values is apparent, without an increase in C20:4n-6. In the subset of patients (n = 8) who developed Long-COVID symptoms, levels of C18:2n-6 were reduced by 60% compared to control subject values (Fig. 2). Collectively, these transitions in essential fatty acid balance among and between plasma phospholipids indicate an overall shift toward increased level of C20:4n-6 in surviving COVID-19 infected subjects receiving supplementary oxygen. In both COVID-19 infected subjects and healthy controls, phosphatidylinositol represents approximately 4.6 ± 0.1% and 4.3 ± 0.2% of total plasma phospholipid respectively. For subjects requiring O_2_ or those developing Long-COVID, phosphatidylinositol contained greater C20:4n-6 [(24.1 ± 0.2% and 24.5 ± 0.04%, respectively)] compared to control subjects (22.6 ± 0.3%) and for subjects requiring no O_2_ (23.6 ± 0.3%; p < 0.01).

**Table 1:** Essential fatty acid composition of total plasma phospholipids.

Fatty acid, N	Control, 27	No O_2_, 66	O_2_ required, 151	Long covid, 8	Deaths, 40
18:2n-6	26.2 ± 0.3	21.4 ± 0.4***	20.6 ± 0.3***	22.5 ± 0.6***	21.0 ± 0.5***
20:4n-6	9.6 ± 0.3	10.9 ± 0.4*	11.4 ± 0.3**	12.5 ± 0.6**	10.5 ± 0.5
18:3n-3	0.66 ± 0.04	0.61 ± 0.03	0.6 ± 0.02	0.38 ± 0.02	0.56 ± 0.03
20:5n-3	0.7 ± 0.07	0.7 ± 0.07	0.5 ± 0.04	0.6 ± 0.04**	0.5 ± 0.06
22:6n-3	1.5 ± 0.1	1.6 ± 0.1	1.8 ± 0.1	2.1 ± 0.03**	1.7 ± 0.1
^A^Inflammatory index	2.8 ± 0.1	2.2 ± 0.1***	2.1 ± 0.1***	1.8 ± 0.1***	2.2 ± 0.2**

Plasma values for COVID-19 infected subjects on hospital admission are compared with control subject values.

Values (mean ± SE) for fatty acid composition are given as %w/w of all fatty acids found in the total plasma phospholipids. Comparisons between COVID-19 infected subjects and healthy control subjects were determined by analysis of variance and are denoted with asterisks (*p < 0.02, **p < 0.01, ***p < 0.001).

^A^Inflammatory index: 18:2n-6/20:4n-6.

Examination of individual phospholipid fatty acid species [(45, 59, 40 and 20 unique species for phosphatidylcholine, phosphatidylethanolamine, phosphatidylserine and phosphatidylinositol, respectively) that quantitatively account for differences in plasma C18:2n-6 and C20:4n-6 in total plasma phospholipids], indicate a shift in essential fatty acid species of phosphatidylcholine and phosphatidylethanolamine for COVID-19 infected subjects compared to control subjects (Table 2). COVID-19 infection results in a remarkable difference in fatty acid species of phosphatidylcholine and phosphatidylethanolamine containing C18:2n-6, C20:4n-6 and C22:6n-3 (Table 2). For example, in phosphatidylcholine, COVID-19 infected subjects have higher 16:0_20:4 species and lower 18:0_18:2 species, and in phosphatidylethanolamine, higher 16:0_20:4, 18:0_20:4 and lower 18:0_18:2 species is observed compared to control subjects. In phosphatidylserine, COVID-19 infection lowers the plasma content of 14:0_18:2 and 16:1_18:2 and raises the content of 18:0_20:4, while markedly decreasing (note fourfold difference) the content of 20:4_18:2 species.

**Table 2:** Essential fatty acid containing species of plasma phospholipids.

Phospholipid fatty acid species, N	Control, 27	No O_2_, 66	O_2_ required, 151	Deaths, 40
Phosphatidylcholine				
16:0_18:2	30.0 ± 0.4	27.9 ± 0.4	29.1 ± 0.3	29.5 ± 0.5
18:0_18:2	12.4 ± 0.2	10.2 ± 0.3	9.5 ± 0.2***	9.3 ± 0.3***
16:0_20:4	6.9 ± 0.3	8.8 ± 0.4**	9.2 ± 0.2***	8.9 ± 0.5**
18:0_20:4	6.7 ± 1.0	8.7 ± 0.4***	7.2 ± 1.7	7.1 ± 0.3
16:0_22:6	1.5 ± 0.1	1.7 ± 0.1	1.9 ± 0.1**	1.9 ± 0.1
18:0_22:6	0.9 ± 0.1	1.0 ± 0.05	1.1 ± 0.03	1.0 ± 0.1
Phosphatidylethanolamine				
16:0_18:2	9.9 ± 0.4	11.1 ± 0.4	11.2 ± 0.2*	11.2 ± 0.6
18:0_18:2	15.3 ± 0.5	12.2 ± 0.5***	11.4 ± 0.4***	10.8 ± 0.7***
16:0_20:4	8.1 ± 0.2	9.2 ± 0.3^a^**	10.1 ± 0.2^b^***	9.5 ± 0.4^ab^*
18:0_20:4	16.5 ± 0.6	18.2 ± 0.5	19.1 ± 0.3*	17.9 ± 0.4
16:0_22:6	6.0 ± 0.3	6.7 ± 0.3^a^	7.7 ± 0.2**^b^	7.2 ± 0.5^ab^
18:0_22:6	2.6 ± 0.2	2.8 ± 0.2	3.3 ± 0.1**	3.1 ± 0.2
Phosphatidylserine				
14:0_18:2	13.3 ± 1.6	4.3 ± 0.6***	13.3 ± 1.6	5.0 ± 0.9***
16:1_18:2	13.4 ± 1.6	4.8 ± 0.6***	4.9 ± 0.4***	5.2 ± 1.0***
16:1_20:4	12.9 ± 2.0	6.7 ± 0.7***	7.3 ± 0.5***	7.6 ± 1.0**
18:0_20:4	5.3 ± 1.6	22.4 ± 1.2***	22.9 ± 0.9***	21.4 ± 1.4***
20:4_18:2	17.5 ± 2.8	5.0 ± 0.7***	4.3 ± 0.6***	5.2 ± 1.0***
16:1_22:6	0.5 ± 0.3	0.8 ± 0.1	0.8 ± 0.6	0.7 ± 0.2
20:4_22:6	1.5 ± 0.6	0.5 ± 0.1	1.0 ± 0.2	1.3 ± 0.3

Plasma values for COVID-19 infected subjects on hospital admission are compared with control subject values.

Values (mean ± SE) for fatty acid composition are given as %w/w of all fatty acids found in each total plasma phospholipid. Comparisons between COVID-19 infected subjects and healthy control subjects were determined by analysis of variance and are denoted with asterisks (*p < 0.02, **p < 0.01, ***p < 0.001). Differences between COVID-19 infected groups are denoted as superscripts (a vs. b; p < 0.01) and were determined after a significant analysis of variance by unpaired t-test.

After COVID-19 infection 16:1_22:6 and 20:4_22:6 species of phosphatidylserine suggests dynamic change within essential fatty acid species of phosphatidylserine following infection. Collectively these quantitative results could be attributed to increased utilization of n-6 fatty acid necessary for viral replication and a need for synthesis of C20:4n-6 and C22:6n-3 from precursor fatty acids to provide more substrate for production of clinically significant levels of proinflammatory eicosanoids after infection.

**Figure 3:**
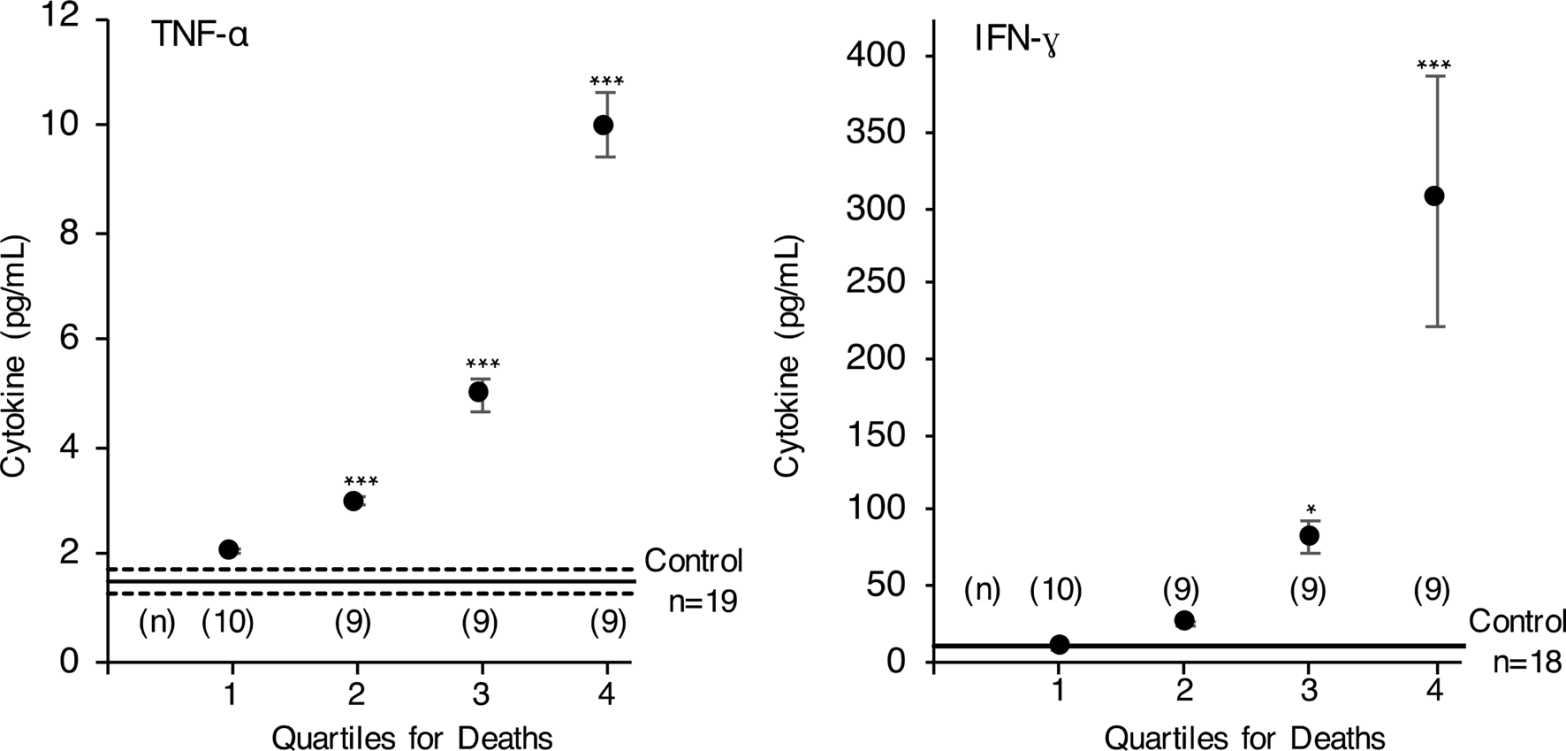
TNF-α and IFN-ɣ levels in plasma on hospital admission for COVID-19 infected subjects who died. Values are expressed as pg of cytokine/ml of plasma. Mean (–) ± SE (–---) is illustrated for healthy control subjects. Significant differences compared to control subjects were determined by analysis of variance are indicated as; *p < 0.02, ***p < 0.001.

Plasma cytokines and chemokines were measured to assess which signals are associated with a potentially proinflammatory fatty acid balance in surviving COVID-19 infected subjects. The plasma level of IFN-γ, IL-10, IL-6, IL-8, TNF-α, eotaxin, MCP-4, MIP-1α and TARC were significantly higher in subjects with COVID-19 infection compared to healthy controls (Table 3). No clear statistical relationship between plasma phospholipid fatty acids and eotaxin, MCP-1, MDC, MIP-1β was found. Levels of IL-8 (tenfold higher in COVID-19 infected patients) and TNF-α were found to be associated with level of C20:4n-6 in subjects who survived infection. For half of the subjects who died, plasma levels of TNF-α and IFN-ɣ are low compared to subjects who survive infection (Table 3 and Fig. 3).

**Table 3:** Plasma cytokine and chemokine levels.

Subject group, N	Control, 20	No O_2_, 65	O_2_ required, 150	Deaths, 40
INF γ	9.3 ± 1.4	120 ± 71***	46 ± 8.7***	104 ± 29*
IL-10	0.4 ± 0.06	4.5 ± 1.5	6.6 ± 2.3**	7.5 ± 1.2***
IL-6	0.6 ± 0.06	43 ± 14***	9.1 ± 1.8***	300 ± 120*
IL-8	2.9 ± 0.2^a^	32 ± 12***^a^	16 ± 1.7***^b^	100 ± 81^ab^
TNF-α	1.6 ± 0.2	4.1 ± 0.5**	3.9 ± 0.5***	5.2 ± 0.5***
Eotaxin	210 ± 27	470 ± 48**	410 ± 35***	490 ± 41***
MCP-1	81 ± 6.6	280 ± 40	170 ± 15	570 ± 180
MCP-4	82 ± 13	210 ± 26**	190 ± 29**	190 ± 24***
MDC	900 ± 170	1100 ± 120	920 ± 101	800 ± 86
MIP-1α	16 ± 2.2	32 ± 2.6	29 ± 2.5*	41 ± 4.8***
MIP-1β	57 ± 7.8	150 ± 21	110 ± 13	130 ± 17**
TARC	60 ± 21	320 ± 49**	310 ± 95*	220 ± 40***

Plasma values (mean ± SE) for COVID-19 infected subjects on hospital admission are compared with control subject values Significant comparisons between COVID-19 infected subjects and healthy control subjects are determined by analysis of variance and denoted with asterisks (*p < 0.02, **p < 0.01, ***p < 0.001). Differences between COVID-19 infected groups are denoted as superscripts (a vs. b; p < 0.01) and were determined after a significant analysis of variance by unpaired t-test. Values are expressed as pg/ml of plasma.

## Discussion

The present study documents that patients who survive COVID-19 infection have higher C20:4n-6 level in plasma phospholipids after infection, likely attributed at least in part to synthesis from C18:2n-6. Higher C20:4n-6 is accompanied by lower levels of C18:2n-6 observed in phosphatidylcholine, phosphatidylethanolamine and phosphatidylserine. Higher content of C20:4n-6 in plasma phosphatidylcholine would be expected to alter balance between n-6 and n-3 fatty acids available for enhanced synthesis of clinically important proinflammatory prostaglandins (E_2_ and A_2_) and leukotrienes (LT B_4_, C_4_, D_4_ and E_4_) altering leukocyte chemotaxis and cell interactions. Elevated production of C20:4n-6 related endocannabinoids and lower production of pro-resolution resolvins and protectins regulate resolution of inflammatory responses^15^. The net result is enhanced secondary production of inflammatory cytokines IL-6, IL-8 and TNF-α and increased cell reactivity in surviving patients. For half of those who died of COVID-19 infection much lower TNF-α and IFN-ɣ levels suggest no or low activation of host immunity in these subjects.

Surviving COVID-19 infected patients had elevated levels of many cytokines measured compared to healthy subjects. The classic proinflammatory cytokines, IL-6, IL-8 and TNF-α have been shown to be strong and independent predictors of patient survival^16^. IL-6 is upregulated by activation of NF-κB, upon binding of coronavirus spike protein to lung epithelial cells^17^ and is the target of recent therapeutics. IL-6 stimulates acute phase macrophage responses, enhances B cell growth and antagonizes T cell responses to infection. IL-8 is an important activator of neutrophils, and a contributor to acute respiratory distress syndrome. The importance of neutrophils during COVID infection has been recently indicated^18^. Increases in cellular responses enhance secretion of TNF-α by macrophage, NK cells and lymphocytes promoting insulin resistance on a cellular level, a pyrogenic response and enhanced inflammation. A counteracting homeostatic inflammatory response by increased IL-10 levels was observed (Table 3). Increase in MIP-1α after infection is indicative of virus induced inflammation. Collectively, these cytokines and chemokines signal proinflammatory change in a cascade of complex metabolic events initiated by alteration in the essential fatty acid balance available from plasma phospholipids that enables synthesis of eicosanoids at sites of uptake by infected cells.

In at least half of the patients who will die of COVID-19 infection, the amount of C18:2n-6 and C20:4n-6 in plasma phospholipid is very low, compared to normal subjects or compared to subjects who survive COVID-19 infection. We propose that this remodelling of plasma essential fatty acid metabolism by the virus rapidly creates a situation for some individuals where the level of these essential fatty acids becomes insufficient (Fig. 1) to enable activation of host immunity and several necessary activating signals such as TNF-α and IFN-ɣ (Fig. 3).

What previously unexpected factors may define the initial severity of disease response to COVID-19 infection? Levels of C20:4n-6 and C22:6n-3 in plasma phospholipids are affected by dietary intake^19^ and by genetic control determined by activity of fatty acid desaturase (FADS1 and FADS2) genes^20,21^. Variants of FADS1 and FADS2 genes have different rates of synthesis of C20:4n-6 and C22:6n-3 and thus genotype affects plasma phospholipid response to essential fatty acid utilization and cell membrane phospholipid levels of C20:4n-6 and C22:6n-3^20^. Observations of the present study raise the question of whether FADS-1 and 2 genotypes that enable enhanced synthesis of C20:4n-6 are also at greater risk for severity of the immune proinflammatory cascade that follows COVID-19 infection. In contrast, are subjects with low capacity for synthesis of C20:4n-6 at risk of not being able to rapidly synthesize sufficient 20:4n-6 to stimulate host immunity needed to initially contain viral replication and the deleterious signals that emanate from infected epithelial cells?

Lipids from the host play a critical role in viral propagation as coronaviruses infect the epithelium, taking over cellular metabolism to support viral replication by catabolizing plasma and intracellular membrane phospholipid of host cells to generate a lipid viral coat^14^. It is noteworthy in the context of the present study’s observations on essential fatty acids that structural analysis of coronavirus spike protein reveals that 18:2n-6 populates the spike protein binding site to effect a change in spike protein confirmation. Each virus contains many spike protein structures, suggesting high utilization of 18:2n-6 by the virus during rapid viral replication^24^.

The conclusions of this study are based on a group of COVID-19 infected patients admitted to hospital for treatment of potentially serious symptoms of infection. COVID-19 infected subjects assessed as not sick enough to require treatment in hospital were not studied. These observations in the present study are limited to those with more severe illness and does not include elderly people in nursing homes at high risk who may not have been sent to hospital. Approximately one in five patients who were studied died of this deadly viral infection.

## Methods

### Ethics and subject recruitment

Ethical approval for the study was obtained from the Human Ethics Committee at the University of Alberta (Pro00100578). The study was performed in accordance with relevant guidelines and regulations. Unvaccinated COVID-19 infected patients (patients infected with SARS-CoV-2) admitted to the University of Alberta Hospital between July 2020 and Jan 2022 provided written informed consent and a blood sample within one day of admission to hospital for symptoms of COVID-19 infection. Blood was collected and plasma separated by the Canadian Biosample Repository (University of Alberta) and stored at −80 °C. Technical and analytical staff were blinded to sample identity and groupings. The statistical assistant was blinded to the identity of subject groupings.

Subject clinical data and descriptive information was accessed through the patient electronic record in Alberta Health Services. COVID-19 infected patients (n = 217) were grouped into those requiring supplemental oxygen (O_2_) to maintain blood oxygen level at greater than 92% (O_2_: n = 151; 79 males, 72 females, age 61.2 ± 1.4 years), those subjects not requiring oxygen (no O_2_: n = 66, 29 males, 37 females, age 55 ± 2.1 years), those subjects who died n = 40 (32 males, 8 females, age 66.9 ± 2.3 years) and those subjects who developed Long-COVID (n = 8, 5 males, 3 females, age 60.1 ± 3.9 years). The number of comorbidities identified for each subject were obtained for COVID-19 infected subjects (1.3 ± 0.1 comorbidities for O_2_ group, 1.5 ± 0.2 comorbidities for no O_2_ group and 0.7 ± 0.6 comorbidities for the Long-COVID group) and for those who died (1.7 ± 0.3) from the hospital electronic records. A group of healthy subjects (age range from 24 to 54 years of age), having no comorbidities (n = 27) and reflecting a wide range of subject ages was also recruited to provide a reference range of normal response for comparison.

### Lipidomic analysis of plasma phospholipids

Plasma lipids were extracted^22^. Plasma (20 μL) was mixed with 400 μL chloroform/methanol (2:1 v/v) containing deuterated lipid internal standard (Equisplash™ Lipidomix^®^ Quantitative Mass Spec Internal Standard, Avanti Polar Lipids, Alabaster, AL, USA) and shaken for 30 min. CaCl_2_ (100 μL of 0.025% w/v) was added and shaken for 30 min. Samples were sonicated, centrifuged and the bottom layer recovered and concentrated under N2. Samples were reconstituted with mobile phase for quantitative lipidomic analysis of individual fatty acid species of each plasma phospholipid^21,23^. Phospholipid extracts were separated by normal phase chromatography (Agilent Zorbax RX-Sil column 3.0 × 100 mm, 1.8 µm particle size) using an Agilent 1260 Infinity LC system (Santa Clara, CA). The total LC run time was 38 min at a flow rate of 0.3 ml/min. Mobile phase A contained isopropanol/hexane/water (58:40:2 v/v/v) with 5 mM ammonium acetate and 0.1% (w/v) acetic acid. Mobile phase B consisted of isopropanol/hexane/water (50:40:10 v/v/v) with 5 mM ammonium acetate and 0.1% acetic acid. Gradient elution consisted of increase in mobile phase B from 34 to 36 min to 100% of mobile phase B. After 36 min mobile phase B was decreased to 0% for 2 more min. Phospholipid species were identified with retention time and internal standard using Equisplash™ Lipidomix^®^ Quantitative Mass Spec Internal Standard. Plasma content was calculated using internal standard and mass spec abundance in the linear range of response.

The Agilent 6430 triple quad LCMS system was operated in multiple reaction monitoring in negative mode^21^. A library of theoretical precursor ions was generated for phosphatidylcholine, phosphatidylethanolamine, phosphatidylinositol, phosphatidylserine or phosphatidylglycerol of varying fatty acid carbon chain length. The product ion was determined by the *m/z* fatty acid composition. Fatty acids were found around the mass region of *m/z* 200–330 according to the deprotonated fatty acid species between 16:0 and 22:6 carbon chain length scanned. The transitions scanned are [M + OAc]¯^→^[FA-H]¯ for phosphatidylcholine; [M-H]¯^→^[FA-H]¯ for phosphatidylethanolamine, phosphatidylinositol, phosphatidylserine and phosphatidylglycerol (see Supplementary Table S1 online). The collision voltage was varied from 18 to 30 eV. FData acquisition and analysis was carried out using Agilent Mass Hunter software.

Quantitative values for plasma content (µg/ml of plasma) of each fatty acid species for each phospholipid were also converted to percent of each fatty acid observed in each phospholipid. Values (% w/w) for fatty acids C18:2n-6, C20:4n-6, C20:5n-3 and C22:6n-3 are illustrated for total plasma phospholipids (Table 1) and for individual phospholipid molecular species (Table 2).

### Cytokine and chemokines analysis

Plasma concentrationsof cytokines and chemokines known to be altered after COVID-19 infection [IL-6, IL-2, IL-8, G-CSF, IFN-ɣ, MCP-1, MIP1-α, TNF-α and thymus and activation-regulated chemokine (TARC)] were quantified using commercially available V-plex Panel kits obtained from Meso Scale Discovery and prepared according to the manufacturer’s instructions.

### Statistical analysis

All data illustrated represent the mean ± standard error. Total of quantitative values for plasma phospholipid C18:2n-6 and C20:4n-6 are illustrated (Fig. 1). An inflammatory index (ratio of C18:2n-6 to C20:4n-6) was calculated to reflect relative changes in balance observed between the precursor (C18:2n-6) and proinflammatory fatty acid (C20:4n-6). One-way analysis of variance using IBM SPSS Statistics version 29 was used to compare values of COVID-19 infected subjects with normal values observed for the range of control subjects recruited. Significant difference between COVID-19 infected subjects given oxygen or not is indicated (a vs b, p < 0.01).

A log transformation was made for individual cytokine values before analysis of variance was used to determine change from normal (control) values (Table 3). Simple correlation between cytokine values, the plasma phospholipid level of C20:4n-6 and inflammatory index was also made.

### Supplementary Information

Supplementary Table S1.

## Data Availability

The datasets used and/or analysed during the current study are available from the corresponding author on reasonable request. Data sharing is not applicable to this article as no datasets were generated or analysed during the current study.
