# Supplementary material for: Plasma essential fatty acid on hospital admission is a marker of COVID-19 disease severity
Source: Sci Rep. 2023 Nov 3;13:18973. doi: 10.1038/s41598-023-46247-0 (PMC10624896; doi:10.1038/s41598-023-46247-0)
Supplement: Supplementary file 1 — Supplementary Table S1. [file 41598_2023_46247_MOESM1_ESM.docx]

**Supplementary Information**

**Supplementary Table S1:** Retention times and MRM transitions from molecular to fragment ion for phospholipid species reported in the manuscript.

| Phospholipid  Species | Precursor  Ion | Product  Ion | Transtion | Retention  Time (min) | Collision  Energy |
| --- | --- | --- | --- | --- | --- |
| Phosphatidylcholine |  |  | [M+OAc]¯^→^[FA-H]¯ |  | 25 |
| PC 16:0_18:2 | 816.6 | 279.2 |  | 32.8 |  |
| PC 18:0_18:2 | 844.6 | 279.2 |  | 32.4 |  |
| PC 16:0_20:4 | 840.6 | 303.2 |  | 32.5 |  |
| PC 18:0_20:4 | 868.6 | 303.2 |  | 32.0 |  |
| PC 16:0_22:6 | 864.6 | 327.2 |  | 32.5 |  |
| PC 18:0_22:6 | 892.6 | 327.2 |  | 32.1 |  |
|  |  |  |  |  |  |
| Phosphatidylethanolamine |  |  | [M-H]¯^→^[FA-H]¯ |  | 18 |
| PE 16:0_18:2 | 714.5 | 279.2 |  | 17.3 |  |
| PE 18:0_18:2 | 742.5 | 279.2 |  | 17.0 |  |
| PE 16:0_20:4 | 738.5 | 303.2 |  | 16.9 |  |
| PE 18:0_20:4 | 766.5 | 303.2 |  | 16.5 |  |
| PE 16:0_22:6 | 762.5 | 327.2 |  | 16.9 |  |
| PE 18:0_22:6 | 790.5 | 327.2 |  | 16.6 |  |
|  |  |  |  |  |  |
| Phosphatidylserine |  |  | [M-H]¯^→^[FA-H]¯ |  | 30 |
| PS 14:0_18:2 | 730.5 | 279.2 |  | 24.0 |  |
| PS 16:1_18:2 | 756.5 | 279.2 |  | 22.6 |  |
| PS 16:1_20:4 | 780.5 | 303.2 |  | 22.3 |  |
| PS 18:0_20:4 | 810.5 | 303.2 |  | 21.7 |  |
| PS 20:4_18:2 | 806.5 | 279.2 |  | 23.9 |  |
| PS 16:1_22:6 | 804.5 | 327.2 |  | 22.3 |  |
| PS 20:4_22:6 | 854.5 | 327.2 |  | 23.7 |  |
